# Microplastics in gastrointestinal tracts of gentoo penguin (*Pygoscelis papua*) chicks on King George Island, Antarctica

**DOI:** 10.1038/s41598-023-39844-6

**Published:** 2023-08-10

**Authors:** Youmin Kim, Hankyu Kim, Min-Su Jeong, Dowoon Kim, Juyang Kim, Jaehak Jung, Hae-Min Seo, Hyun-Jin Han, Woo-Shin Lee, Chang-Yong Choi

**Affiliations:** 1 Department of Agriculture, Forestry, and Bioresources, Seoul National University, Seoul, 08826 Republic of Korea; 2 Division of Life Sciences, Korea Polar Research Institute, Incheon, 21990 Republic of Korea; 3 Korea Institute of Analytical Science and Technology, Seoul, 04790 Republic of Korea; 4 Taxidermy Lab, Icheon, Gyeonggi 17402 Republic of Korea; 5 Research Institute of Agriculture and Life Sciences, Seoul National University, Seoul, 08826 Republic of Korea; 6 Present Address: Department of Forest and Wildlife Ecology, University of Wisconsin-Madison, Madison, WI 53706 USA

**Keywords:** Ecology, Zoology, Environmental sciences, Ocean sciences

## Abstract

Microplastics (< 5 mm) have been found in marine ecosystems worldwide, even in Antarctic ecosystems. In this study, the stomach and upper intestines of 14 dead gentoo penguin (*Pygoscelis papua*) chicks were collected and screened for microplastics on King George Island, a gateway to Antarctic research and tourism. A total of 378 microplastics were identified by Fourier-transform infrared spectroscopy, with 27.0 ± 25.3 microplastics per individual. The detected number of microplastics did not increase with the mass of penguin chicks, suggesting no permanent accumulation of microplastics. However, the concentration of microplastics was much higher (9.1 ± 10.8 microplastics per individual within the size range 100–5000 μm) than the previously reported concentration in the penguin feces, and a greater number of smaller microplastics were found. Marine debris surveys near the breeding colony found various plastic (79.3%) to be the most frequent type of beached debris, suggesting that local sources of marine plastic waste could have contributed to microplastic contamination of penguin chicks being fed by parents that forage in nearby seas. This finding confirms the presence of microplastics in an Antarctic ecosystem and suggests the need for stronger waste management in Antarctica and a standardized scheme of microplastic monitoring in this once-pristine ecosystem.

## Introduction

Plastic pollution is causing a serious disturbance in marine ecosystems worldwide^1^. Not only are marine plastic debris widespread^2–5^, but marine microplastics (smaller than 5 mm) have been reported globally^6^ and widely dispersed in marine ecosystems^7–9^, from invertebrates and fishes^10–13^ to seabirds^14–17^ and marine mammals^18–20^. As technical advancements have allowed researchers to analyze even smaller particles, the number of studies on microplastics in marine ecosystems has increased^21^. Marine microplastics originate from anthropogenic sources on land^22–24^ or from macroplastics of the same origin that are broken down in the ocean^2,15,22^. Marine microplastics mainly exist in the form of fibrous materials (microfibers), small fragments, or thin films^25^, and are presumed to be transferred to upper trophic levels through the food web^7,8,26^. The effects of microplastics at the organism level require further investigation^27–31^, but they can negatively affect the health of an organism^28,32^ and act as one of the multiple pollutants in ecosystems^33,34^.

The Antarctic region has been an area relatively free from anthropogenic disturbance and pollution because human activities are restricted by the Antarctic Treaty, and the Madrid Protocol refers to waste disposal and prevention of marine pollution in the region^35^. But microplastics have been reported in oceanic fauna from Antarctic marine ecosystems^4,10,36–39^. Atmospheric and oceanic circulation makes the Antarctic region a sink for microplastics transferred from across the globe^36,40^, but direct local input from an increased number of tourists^36^, fishing vessels^41,42^, and research stations^36,38^ also act as both primary and secondary sources of microplastics in the Antarctic marine systems^36^. Therefore, increasing anthropogenic activities are aggravating marine pollution in the region^43^, especially in the Antarctic Peninsula. Previous studies reported a few plastic marine debris items from research stations in the Antarctic Peninsula region^44,45^, but plastics were found in gentoo penguin (*Pygoscelis papua*) stomachs from penguin colonies near stations^17^. The density of marine microplastics accumulating in the environment^39,46^ and organisms including zooplankton^37^ and top predators^47,48^ is higher in the Antarctic Peninsula than those in other Antarctica regions.

Seabirds represent suitable indicator species of marine pollution^48,49^, including marine plastic pollution^47,48^. While many studies showed microplastic ingestion in seabirds, there have been only a few reports on penguins in Antarctica^47,48,50^. Previous studies investigated plastic ingestion in seabirds through feces, pellets, and samples from gastrointestinal tracts, but the methodology used to detect and quantify microplastics in those studies was not standardized^14,51,52^. Specifically, the target size and type of microplastic vary among studies that investigated microplastic ingestion by large marine predators with different study objectives, survey efforts, and sampling and analytical methods^19,53^. For example, two microplastic ingestion studies of penguins in the genus *Pygoscelis* reported that 15–29% of the feces contained microplastics^47,48^. These previous studies on microplastics in penguins used fecal samples^47,48^ and each study reported only a certain type or size of microplastics (e.g., only microfibers between 186 to 9280 µm were analyzed in king penguins *Aptenodytes patagonicus*^50^). However, fecal analysis can only provide information on microplastics successfully discharged through the digestive system of penguins, making it difficult to determine the amount and type of microplastics that are actually ingested by or retained in individuals.

To bridge the current knowledge gap on microplastics in a marine ecosystem from the region, we investigated microplastics accumulated inside the body of *Pygoscelis* penguins and local marine debris composition in an Antarctic marine ecosystem. We aimed to assess the quantity and qualitative characteristics of microplastics ingested by gentoo penguin chicks based on gastrointestinal tract samples in this study. Given the high human activities on King George Island in the South Shetland Islands, we hypothesized that more and smaller microplastics would be detected in gentoo penguin chicks than in previous reports because they might adhere more easily to the mucosal lining of the gastrointestinal tract. Using Fourier-transform infrared (FT-IR) microscopy and a nano-sized filter, we screened for a wider size range of microplastics and identified different types of plastics from the samples. With these data, we described the composition of microplastics ingested by gentoo penguin chicks and compared our results with existing reports of plastic ingestion in Antarctic penguin species using different sampling techniques. In addition, we performed marine debris surveys around the study area to obtain a snapshot of marine plastic pollution and secondary microplastic sources to explore the potential influence of local inputs on microplastics in Antarctic ecosystems.

## Results

### Marine debris monitoring

**Figure 1:**
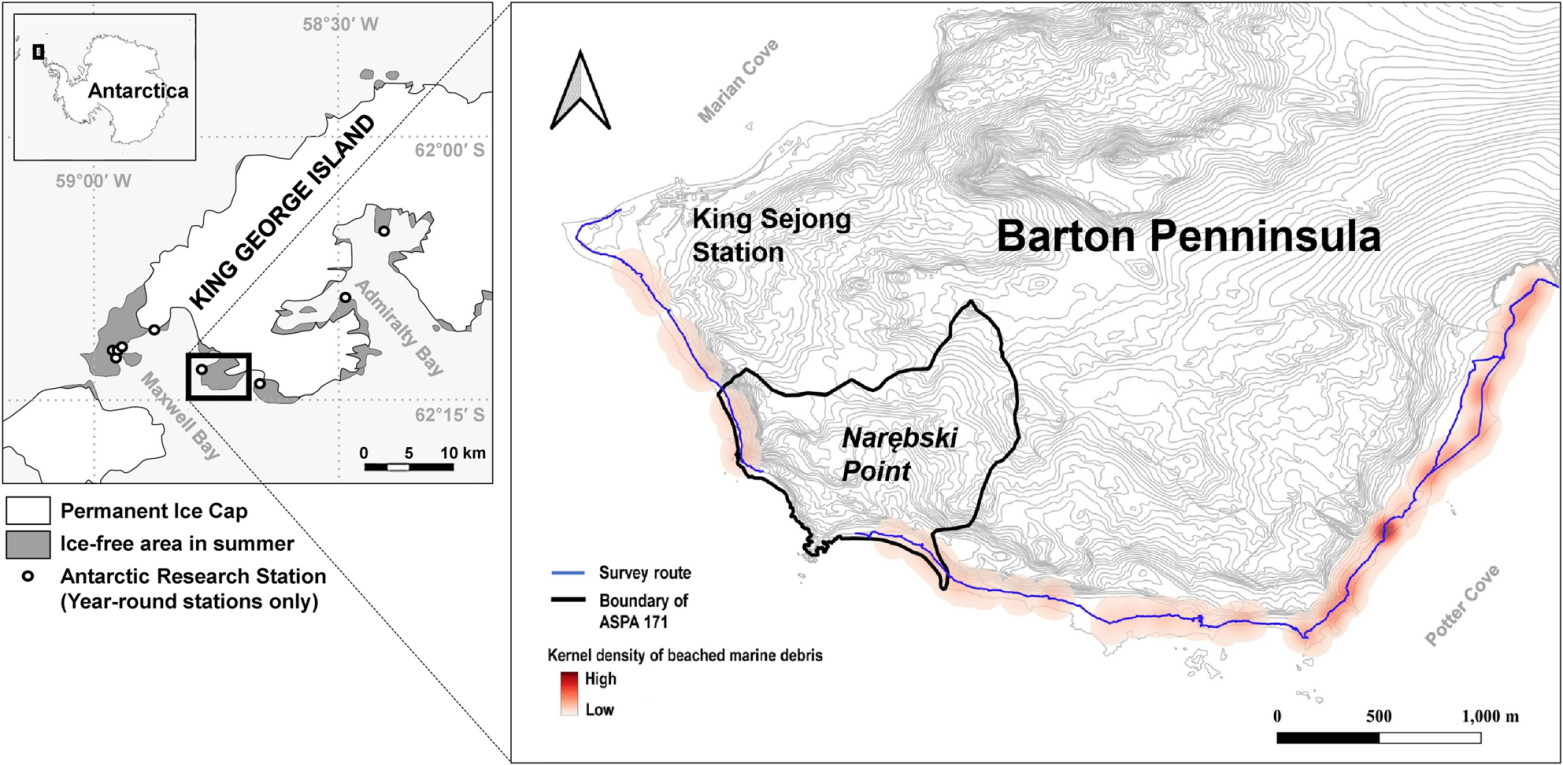
The distribution of beached marine debris on the Barton Peninsula of King George Island, South Shetland Islands, Antarctica. The boundary of Narębski Point (Antarctic Specially Protected Area 171: ASPA 171), where the penguin colonies are located, was marked by an orange line, and the kernel density of marine debris was shown as a red gradient. The year-round Antarctic Research Stations on King George Island were marked by circles in the index map. This map was created in ArcMap 10.2.2 from base map data in the public domain (the polar geographic information; http://map.ngii.go.kr/) provided by the Korean Government.

A total of 151 beached marine debris were found in five field surveys during the summer of 2013/14 and 2014/15. Most were plastic (79.30%), while some were metal (7.86%) and other materials (12.84%) (Table 1, Table S1). The specific type of debris included small unidentified plastic fragments (41), ropes (24), polystyrene (24), polyurethane insulation foam (15), and cargo box debris (10) (Fig. S1, Table S1). Smaller and lighter debris were found more frequently, and 61.6% weighed less than 0.01 kg. The spatial density of surveyed marine debris was high on the southeast coast of the Barton Peninsula, but marine debris were found along the entire survey route (Fig. 1).

**Table 1:** Composition of marine debris found during five surveys in the summer of 2013/14 and 2014/15 on the Barton Peninsula of King George Island, Antarctica.

Description	Material	Mass (kg)					%
		< 0.01	0.01–0.1	0.1–0.5	0.5–1.0	> 1.0	
Rope	Plastic	11	5	4	2	2	9.30
Polyurethane foam	Plastic	13	2				10.71
Plastic fragment	Plastic	18	5	8	7	3	29.30
Plastic bottle	Plastic	4	3	1			5.71
Polystyrene	Plastic	22	1		1		17.14
Cargo debris	Plastic	7	1	1	1		7.14
Metal wire	Metal	1	1	1	2		3.57
Metal fragment	Metal	1	1	1	1	2	4.29
Rubber fragment	Rubber		1				0.71
Tile fragment	Ceramic	1					0.71
Float fragment	Glass			1			0.71
Clothing fragment	Others	1					0.71
Paper	Paper	5					3.57
Wood fragment	Wood	1					0.71
Food	Others	8					5.72

### Fourier-transform infrared analysis of gastrointestinal tract samples.

**Figure 2:**
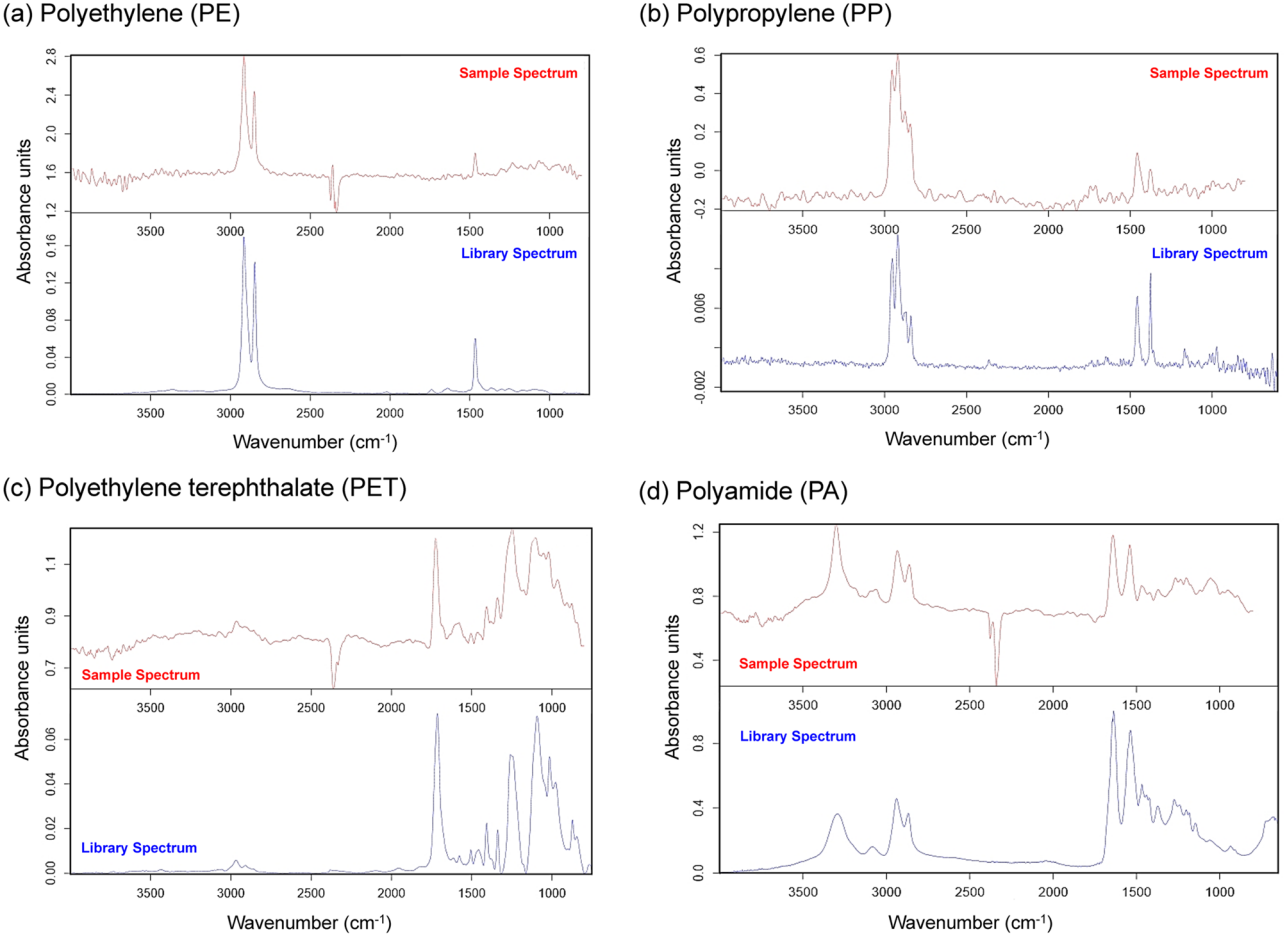
The Fourier Transform Infrared (FT-IR) spectra of microplastics found in the gastrointestinal wall and lining of gentoo penguin (*Pygoscelis papua*) chicks: (**a**) polyethylene (PE), (**b**) polypropylene (PP), (**c**) polyethylene terephthalate (PET), and (**d**) polyamide (PA). The spectra of the sample and library of each microplastic type were shown in the upper and lower panels, respectively.

**Figure 3:**
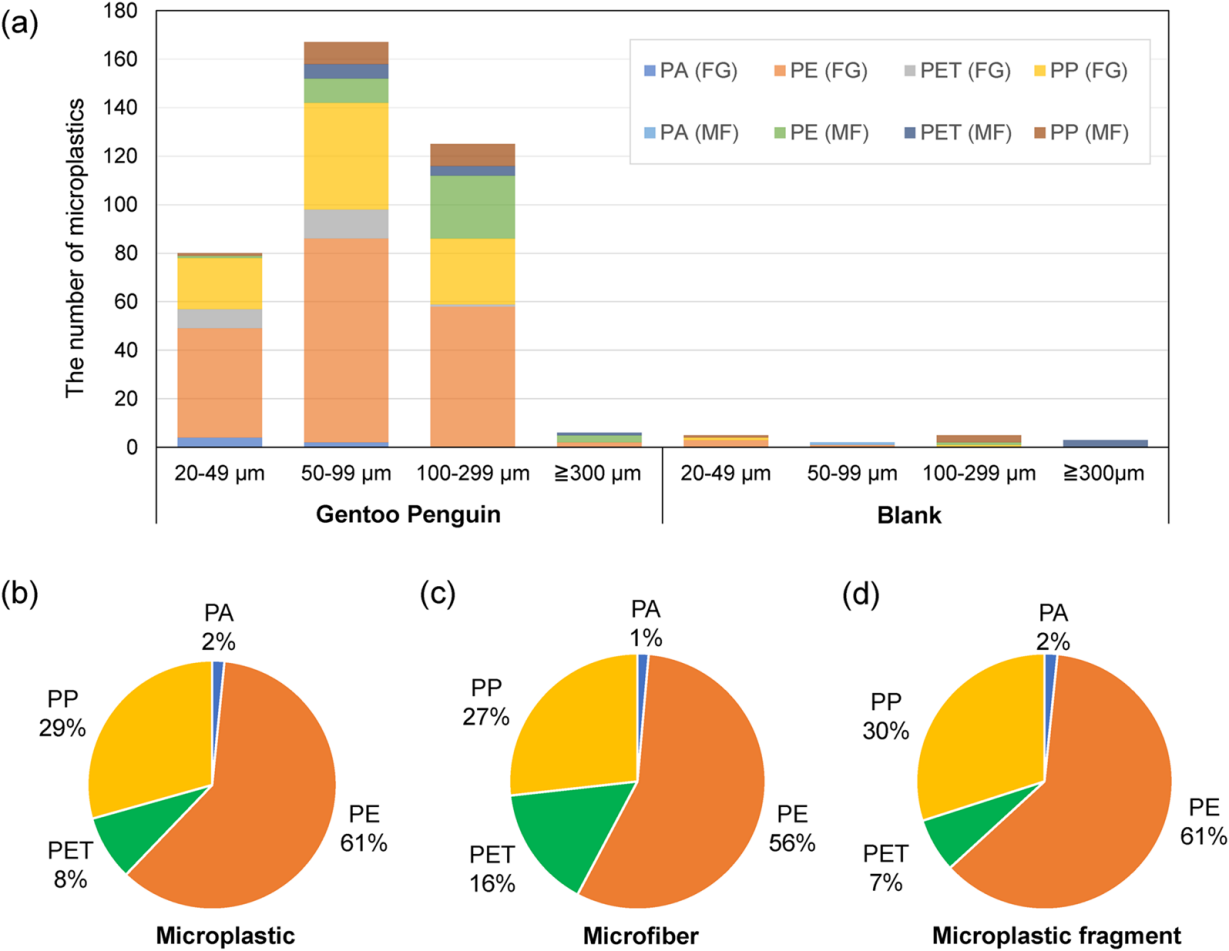
The microplastics detected in the gastrointestinal wall and lining of gentoo penguin (*Pygoscelis papua*) chicks. (**a**) The number of microplastics by size, form, and type in samples (noted as Gentoo Penguin) and controls (noted as Blank). The microplastics were found in two different forms, mostly fragments (FG; 81.48%) and a few microfibers (MF; 18.52%), and the four plastic types detected were polyamide (PA; n = 6), polyethylene (PE; n = 229), polyethylene terephthalate (PET; n = 32), and polypropylene (PP; n = 111). The plastic types were presented in (**b**) all microplastics, (**c**) microfibers, and (**d**) fragments. See Table 2 for exact number of microplastics found in each samples.

Dried mass of stomach samples from each carcass was 2.03 ± 0.37 g (range: 1.37–2.84 g) on average, and a total of 378 microplastics of four different types (Fig. S2) were found from 14 gentoo penguin chicks (Tables 2 and 3 and S2, Figs. 2 and 3). Per individual bird, we found an average of 27.00 ± 25.28 (2–81) microplastics. On the other hand, in the five control blanks, only 11 microplastics were detected (1.80 ± 1.25 microplastics per sample, n = 5, Fig. 3). The mean estimated age of chicks was 21 day-old (ranging from 12 to 26 day-old; Table 2), and the generalized linear regression between the number of microplastics per unit sample mass and the body mass of chicks was not significant (n = 14; total body mass with prey: coefficient = 0.00042, *P* = 0.269, body mass without prey: coefficient = 0.00048, *P* = 0.353; Fig. S4).

**Table 2:** The number of detected microplastics in the gastrointestinal wall and lining of gentoo penguin (*Pygoscelis papua*) chicks.

Sex of penguin chicks	Total body mass of penguin chicks (g)	Body mass of penguin chicks without prey (g)	Estimated age of penguin chicks (day)	Dried sample mass (g)	The number of overall microplastics (the number of microfiber)				
					Polyethylene	Polypropylene	Polyethylene terephthalate	Polyamide	Total
F	547	473	12	1.891	2 (1)				2 (1)
M	702	592	16	1.373	35 (4)		6 (1)	1	42 (5)
M	742	729	15	1.983	14 (6)		1		15 (6)
F	818	671	17	2.144	3	27 (3)	7 (2)		37 (5)
M	839	827	18	2.117	5	1	2		8
F	1389	1286	23	2.084	8	5			13
U	1598	1460	23	2.313	5 (1)	1			6 (1)
M	1,628	1,138	20	2.429	8 (2)	2	5 (5)		15 (7)
M	1896	1451	25	2.059	15 (1)	59 (15)		1	75 (16)
F	1,980	1,496	24	1.949	9 (1)	1			10 (1)
M	2121	1653	25	1.908	6		1	2	9
M	2155	1708	23	2.84	21 (5)	1	1 (1)		23 (6)
M	2256	1807	26	1.381	24 (3)	8	8 (2)	2	42 (5)
M	2416	1877	26	2.001	74 (16)	6 (1)	1		81 (17)

The number of microplastics with fiber type is given in parentheses. All of the carcasses were collected at the gentoo penguin colony (central coordination: 62° 14.16′ S; 58° 46.50′ W) at Narębski Point on King George Island, Antarctica. F, M, and U denote female, male, and unknown sex, respectively.

**Table 3:** Comparison of results from microplastic (MP) ingestion studies in different Antarctic penguin species. The results of this study were shown in the bottom row.

Species	Location	Sample type and size	Microplastic form and size	Total number of microplastics	Microplastics Concentration	Composition of microplastics
King penguin (*Aptenodytes patagonicus*)^50^	Hound Bay, South Georgia	Fecal samples (n = 47)	Microfiber (186–9280 µm)	A total of 13 microfibers	0.28 per feces	6 Polyethylene terephthalate, 5 Acrylic, 2 Polypropylene
Gentoo penguin (*Pygoscelis papua*)^47^	Bird Islands, South Georgia and Signy Island, South Orkney Islands	Fecal samples (n = 80)	All forms of microplastics (76–4945 μm)	A total of 19 MPs (5 fragments, 11 microfibers, and 3 films)	0.23 ± 0.53 per feces	11 Polyethylene terephthalate, 3 Cellulose, and one of each of 5 types
Adelie (*Pygoscelis adeliae*), chinstrap (*P. antarticus*), and gentoo penguins^48^	Bird Island, and 9 colonies near the Antarctic Peninsula	Fecal samples (n = 317)	All forms of microplastics (63–5000 μm)	A total of 92 anthropogenic particles, 72% fibers, and 26% fragments. Only 10 of them are MPs	0.03 per feces	8 Polyethylene, 1 Polyethylene terephthalate, 1 unidentified
Gentoo penguin	Narębski Point, King George Island	Gastrointestinal tissues (stomach lining and upper 30 cm intestinal lining) from 14 chicks	All forms of microplastics (20–5000 μm)	A total of 378 MPs (307 fragments and 71 microfibers)	27.00 ± 25.28 per individual (20–5000 μm), 9.14 ± 10.82 per individual (100–5000 μm)	Polyethylene (61%), Polypropylene (29%), Polyethylene terephthalate (8%), and Polyamide (2%)

Most of the microplastics found in penguin chicks were polyethylene (n = 229, 60.58%) and were in the form of fragments (n = 308, 81.48%) (Table 2 and 3, Fig. 3). The microplastic fragments were detected most frequently in a size order of 50–99 μm (n = 142, 46.11%), 100–299 μm (n = 86, 27.92%), 20–49 μm (n = 78, 25.32%), and 300–5,000 μm (n = 2, 0.65%). The plastic types of microplastic fragments were polyethylene (n = 189, 61.36%), polypropylene (n = 92, 29.87%), polyethylene terephthalate (n = 21, 6.82%), and polyamide (n = 6, 1.95%). On the other hand, the microplastic fibers (microfibers) appeared at a different frequency of size; those were detected most frequently in the range of 100–299 μm (n = 39, 55.71%), followed by 50–99 μm (n = 25, 35.72%), 300–5,000 μm (n = 4, 5.71%), and 20–49 μm (n = 2, 2.86%). The plastic type of microfibers was polyethylene (n = 40, 57.14%), polypropylene (n = 19, 27.14%), and polyethylene terephthalate (n = 11, 15.71%).

## Discussion

Gentoo penguin chicks on King George Island ingested a number of microplastics during the short period after hatching (estimated 12–26 days). There are a few potential pathways for penguin chicks to ingest microplastics, and most microplastics might originate from food items, as in the case of other marine predators^18,26^. In our study area, adult gentoo penguins mainly feed their young with Antarctic krill (*Euphausia superba*)^54,55^, a superabundant marine zooplankton that feeds on planktonic algae^56^. It was already reported that krill^57^, the main prey of Antarctic penguins^58^, ingested microplastics in a laboratory environment. Plastics might be introduced into the sea from research stations^37,38^, fishing and tour activities^2,37^, and by currents^34,40^ to accumulate through Antarctic krill into gentoo penguin chicks^47,57^.

We detected a broader range of microplastics (20–5000 μm), which make us find a greater number of microplastics in penguin compared to other seabird species living in the lower latitudes, where anthropogenic activity is more intense than in the Antarctic region. In previous studies, northern fulmars (*Fulmarus glacialis*; 11.6 ± 21.6; 400–5000 μm) in the Labrador Sea^59^, along with beached northern fulmars (19.5 ± 2.1; detected > 1 mm) and sooty shearwaters (*Ardenna grisea*; 13.3 ± 3.5; detected > 1 mm) on Pacific Ocean beaches^60^, showed lower levels of microplastic concentrations in their digestive. tracts This appears to be due to differences in the size range of the detected microplastics. Though the direct comparison of microplastic pollution with other local seabirds is difficult, our study confirmed that even the chicks of gentoo penguins were exposed to microplastic pollution from nearby seas where parent birds forage (20–5000 μm in size, 27.00 ± 25.28 per individual).

The number of microplastics in our study was much higher than those in previous reports^47,48,50^ on penguin species in Antarctica, even except for those smaller than 100 μm (9.14 ± 10.82 per individual; 100–5000 μm in size; Table 3). In most studies of seabird microplastic intake using fecal samples, microfibers accounted for a high proportion of microplastic particles^15,47,53^. However, we found that 81% of microplastics were in the form of fragments and that 94% of detected microplastics were less than 300 μm in size, which is smaller than most of the reported microplastics in studies of penguin feces^47,50^. These differences seem to be caused by different sampling materials and target microplastic sizes, as we examined the lining of the gastrointestinal tract instead of digested food content. While it is unclear how the digestive tracts of penguins remove indigestible items, larger-sized microfibers or microplastic fragments have been observed to be excreted with feces in other studies^15,53^. We suggest that small and lightweight microplastic particles might remain attached to the intestinal mucosa and surface structures in the gastrointestinal tract, unlike larger micro- and macroplastics that may act in two extreme ways: getting simply entrapped in or easily passing through the digestive tract. On the other hand, in a study on microplastic ingestion in emperor penguin (*Aptenodytes forsteri*) chicks using similar sampling methods and sample types, the microplastic concentrations in the Antarctic continent might still be neglectable as few microplastics were found^61^. However, the emperor penguin study aimed to identify relatively large microplastics (> 500 μm) in gizzards ^61^, whereas this study detected more broad size ranges of microplastics focusing on smaller ones (20–5000 μm) in the upper gastrointestinal tract including gizzards. As we hypothesized, this study detected only two microplastics larger than 500 μm but many more in the smaller size categories, suggesting the high microplastic pollution in our study site. This means that the difference in size categories of microplastics should be considered with caution, in addition to different sampling efforts, methods, and species’ ecological traits. Therefore, the standardization of sampling methods and size categories is crucial for comparing regional microplastic pollution from different studies.

In this study, microplastics were detected in the order of polyethylene, polypropylene, polyethylene terephthalate, and polyamide for both fiber and fragment types (Fig. 3). Polyethylene and polypropylene accounted for 61% and 29% of detected microplastics. Both types of plastic are routinely used in daily life as like plastic bags^62^ and bottle caps^63^, including at Antarctic research stations^36^. Therefore, the high level of microplastic pollution is presumed to be due to high human activities at the study site, including research stations, fishing activity in nearby oceans, and frequent visits by tourist cruises along with possible influence from currents. Microplastics in the ocean could be delivered to gentoo penguin chicks through the local food web, especially through Antarctic krill which is their main food source^57,64,65^. The Antarctic krill is known to consume and contain microplastics due to their filter feeding^57,65^, and the type of microplastics detected in this study showed a similar composition to those found in the krill collected from the nearby ocean where our penguins forage: the most common polyethylene and least common polyamide^65^. Moreover, microplastics in the size range of 50–100 μm accounted for the highest proportion in both krill and penguins^65^. While the mean microplastic item per krill was low^65^, constant provisioning of krill by adult penguins potentially led to a higher number of microplastics in the digestive tracts of their offspring. Nevertheless, this supportive finding from krill does not confirm the source of microplastics in the penguin chicks, because the microplastics both in krill and penguins may be a simple subset of the shared local contamination. Therefore, the main source and pathway of microplastics from the ocean to the penguin chicks remain to be further discovered.

The effect of microplastics on top predators has not been clearly established. Studies on fish showed that microplastics might cause physiological problems or potential negative impacts through interaction with other toxic chemicals^28,66–70^. However, there are reports that microplastics show negligible impacts on wildlife^71,72^. Moreover, microplastics can have different effects on the physiology of animals depending on their chemical structure, composition, and size^73–75^. For example, smaller particles that can be retained longer, like those detected in our analysis, can potentially expose growing penguin chicks to toxicological consequences^76^. Therefore, further research is needed to investigate the impacts of microplastics on wildlife, depending on their type and size.

The number of detected microplastics in our samples did not increase with the body mass of penguin chicks, suggesting that microplastics might not accumulate permanently in the gastrointestinal tracts of penguin chicks. Together with previous studies showing the fecal prevalence and concentration of microplastics, our findings imply that ingested microplastics can be excreted as feces^53^. However, it is still unclear what amount of ingested microplastics are retained in the digestive tract, and for how long^15,53^. Because the turnover rate and retention time of microplastics in penguins’ guts remain unclear, it is difficult to quantitatively compare our results with other studies using fecal samples. A systematic random sampling of fecal matter of adults and chicks may provide an unbiased representative prevalence of microplastic contamination in a population, but it may not reflect the true intensity and concentration of microplastics in avian digestive systems. In addition, even though there is no proven direct relationship between the high concentration of microplastics and increased mortality, the possibility of a biased sample should be considered because we only used dead chicks as a passive and non-invasive sampling scheme. A combination of non-invasive fecal sampling across a breeding colony of seabirds and necropsy sampling of deceased birds could provide better information on microplastic pollution in seabirds and marine ecosystems by supplementing their own limits.

Using gastrointestinal tracts from carcasses can be useful for studying microplastics in top predators, and this could be a non-invasive monitoring method similar to plastic pollution monitoring using beached birds^77^. However, a sufficient number of carcasses and a standard method of sampling are needed. For example, even though we tried to standardize our sampling efforts by scraping the stomach and the upper 30 cm of the intestine, the growth of chicks and the associated change in size and parts of sampling digestive organs are hard to control. Another important issue is to prevent extraneous plastic contamination^52^. Despite the strong preventive measures against contamination we took in this study, such as using non-plastic tools and cleaning glass vials with a filtered solution through a metal filter (5 μm), low levels of microplastics were still detected in the control group, of which 91% were microfibers. These were thought to have been introduced through the air during the sample drying process or from the clothing worn during sampling. Minimization of possible contamination and use of control ‘blank’ samples in each run should be adopted.

We confirmed that a large amount of plastic marine debris was found along nearly all surveyed coastlines of the Barton Peninsula, King George Island, though the distribution was not consistent possibly due to uneven effects of terrains, winds, and waves. Although our study cannot establish clear connections between microplastics found in penguin chicks and the plastic debris on beaches, it is important to note that local beached marine debris can indicate marine plastic pollution in the surrounding marine habitat for the birds. Most of the marine debris was plastic, indicating that the penguins in our study site may not be free from marine plastic pollution as found in their gastrointestinal tract. Unlike previous studies on Scotia Arc, which mostly reported plastic bottles and polystyrene^78^, this study identified a high frequency of insulating foams and cargo debris, which are often used at stations and in vessels. Hard plastic fragments, which are most likely high-density polyethylene, plastic bottles (polyethylene and polyethylene terephthalate), and nautical rope segments (polyamide) were marine debris items that appeared at high frequency and mass, and reflect plastic materials that were commonly detected in the digestive tracts of penguins in our study. We also found a similar amount of beached marine debris in each survey round despite cleaning efforts by researchers and staff, suggesting a high level of turnover due to a high abundance of marine waste. Although we were not able to identify the exact origin of these plastic debris (e.g. building materials, ropes, buoys), many of them likely originated from the nearby research stations and vessels for fisheries, tourism, or transportation given the co-occurrence with abandoned fresh food items (e.g. half-cut lemons, green onions) (Table S1). To control the secondary source of microplastics in Antarctic marine ecosystems, on-site measures to reduce local macro- and microplastic pollutants in target management areas are needed.

Since the presence of plastics in Antarctica has been confirmed, the Antarctic Treaty System (ATS) is proposing plans to reduce marine plastic pollution^43^. To reduce the disposal of plastic debris from fishing vessels, the Commission for the Conservation of Antarctic Marine Living Resources (CCAMLR) adopted Conservation Measure 26–01 (CM 26-01), ‘General Environmental Protection Measure During Fishing’ in 2006^79^, and Resolution 5 (2019), ‘Reducing Plastic Pollution in Antarctica and the Southern Ocean’ was adopted at the Antarctic Treaty Consultative Meeting (ATCM) in 2019. Furthermore, the International Association of Antarctica Tour Operators (IAATO) has guidelines for reducing waste^80^. However, as it is expected that anthropogenic disturbance caused by the increasing number of people visiting Antarctica will continue to increase^81^, there is a demand for further high-level restrictions on human activities in Antarctica^82,83^. In order to resolve the problem of plastic pollution in the Southern Ocean, education and management of visitors are also required with global cooperation. At each research station, a pollution reduction manual based on ATCM is being applied, and appropriate education is provided to researchers and staff^84^. Along with the global voluntary actions and legally-binding agreements like the proposed UN Treaty on Plastic Pollution, it is necessary not only to make continuous efforts to reduce and manage plastic pollution in the Southern Ocean but also to conduct further related research including on pollution sources and continuous monitoring of marine plastics.

Antarctica is a region with relatively low human disturbance so far, but as human activities are increasing for several purposes, microplastic pollution is expected to increase gradually and continuously. This study showed the highest level of microplastic contamination ever reported in Antarctic penguins with advanced detecting technology and a high prevalence of secondary microplastic sources in the vicinity of the Antarctic protected area. However, the main cause of this contamination and its impact on penguins in Antarctica remains unclear. Nevertheless, this study strongly suggests that more comparative studies are needed to develop a standardized protocol, assess the current state, and monitor changes in microplastic pollution in Antarctic ecosystems.

## Methods

### Study site

King George Island is the largest island of the South Shetlands in Antarctica, and it has three major bays: Maxwell Bay, Admiralty Bay, and King George Bay. The first research stations were built in 1968, and now there are 10 stations. Seven of these stations are located around Maxwell Bay, including five on the Fildes Peninsula, one on the Barton Peninsula, and one on the Potter Peninsula (Fig. 1). King George Island has the only airport on the South Shetland Islands and is a central logistic hub for the northern Antarctic Peninsula with the highest level of human activities. For example, during the 2019/20 summer (October–April), a total of 35,471 tourists and 4,486 researchers and station support staff visited King George Island, and these numbers exceeded 50% of total visitors to Antarctica^81^.

The Barton Peninsula is located on the southwestern part of King George Island facing the center of Maxwell Bay. King Sejong Station is situated at the western edge, and Narębski Point (Antarctic Specially Protected Area No. 171) is situated on the southwestern coast of Barton Peninsula. The northwest coast of Barton Peninsula is mostly cliffs and mountains, while the southeast and southwest coasts consist of pebble beaches and steep slopes beyond. Narębski Point covers an area of about 1 km^2^, and 2000 pairs of gentoo and 3000 pairs of chinstrap penguins (*P. antarcticus*) breed there annually^84^.

### Marine debris monitoring

We surveyed beached marine debris along the southwest and southeast coasts of Barton Peninsula around Narębski Point on a survey track 6.5 km in length, from King Sejong Station to the west coastline of Potter Cove (Fig. 1, Fig. S1). We conducted two surveys in 2013/14 (23 December 2013 and 9 January 2014) and three in 2014/15 (22, 30 December 2014 and 11 January 2015). We visually searched for marine waste on the surface along the shoreline, approximately within 15 m from the tide line except for hard-to-reach areas such as cliffs, and searched for beached anthropogenic marine debris items from the tide line where other ‘natural’ debris (kelp and animal carcasses) wash up. We recorded the coordinates with a GPS tracker (Oregon 550, Garmin, USA), described the type of debris, and measured the mass of the found debris. The mass was measured to the nearest 0.01 kg using a spring scale (Pesola, Switzerland) or to 0.1 kg with a digital scale for bigger debris (HS-50 K, Hansung, Korea). All waste was retrieved and taken to King Sejong Station for proper disposal. We describe the composition of found marine debris in number and weight, and the spatial distribution of marine debris is presented as a kernel density plot using ArcMap 10.2.2 (ESRI, USA).

### Sample collection

A total of 39 carcasses of gentoo penguin chicks were collected during long-term monitoring of breeding penguins at Narębeski Point (ASPA 171) in the austral summer of 2018/19 (Fig. S3)^85^. We selected 14 whole carcasses without external injury from nests and stored them in a − 60 °C freezer until they were dissected at the station. Before the dissection, we measured their morphometrics (including body mass and lengths of head, bill, flipper, and foot) as well as the mass of prey in their guts. The body mass of chicks ranged from 547 to 2416 g, and the age was estimated using the measurement data with the known growth curve of gentoo penguin chicks investigated in the same site (Table 2)^85^. This information was later used to determine the linear relationship between the number of found microplastics per sample mass and the penguin’s body mass (with and without prey mass) using generalized linear regression analysis in R 4.1.0^86^. We applied the negative binomial regression model with an offset using the *MASS* package^87^ to control the difference between the mean and variance, especially overdispersion, and the different dried sample masses used for lab analysis.

To analyze the microplastics accumulated in the gastrointestinal tract, we removed the contents of the gizzards and intestines and rinsed visible food remnants with ultrapure water (Milli-Q Direct 16, Merck KGaA, Germany) before sampling. Then the gastrointestinal wall and inner lining of the stomach and the upper 30 cm of the intestine connected to the gizzard (mainly duodenum and jejunum) were scraped with a metal scalpel, and the samples were collected and stored in a glass vial. We used metal or glass tools only for sample collection to avoid any possible plastic contamination, and all metal and glass materials including the vials were also cleaned with ultrapure water before being used. Collected samples were dried for more than 72 h in a dry oven, and the vial was sealed with a rinsed silicon cap and stored at − 20 °C for further analysis.

### Extraction of microplastics

The sealed vials were transported to the Korea Institute of Analytical Science and Technology (KIAST) in Seoul, Korea for lab analysis. Each sample was transferred to a 1-L glass beaker cleaned with a potassium hydroxide (KOH, 10%) solution, and then 200 ml of 10% KOH solution was added and heated to 40 °C on a hot plate for 24 h^88^. After 24 h, a total of 50 ml of hydrogen peroxide (H_2_O_2_, 30%) was added over 3 to 5 days. When adding H_2_O_2_ to the KOH solution, the solution could overflow due to the reaction between H_2_O_2_ and KOH. Therefore, H_2_O_2_ was added in 1–5 ml portions. After three to five days of digestion, the solution in the flask was vacuum -filtered through a 20-μm metal filter (KF-STC2520, KIAST).

To remove any remaining organic matter after KOH digestion, the Fenton reaction was performed with the method in a previous study^89^. Briefly, after filtering the above KOH-digested solution, the metal filter was transferred to a clean 1-L Erlenmeyer flask; 10 ml of iron sulfate heptahydrate (FeSO_4_·7H_2_O, 20 g/L) was added and then 20 ml of H_2_O_2_ was added. Starting one minute after the initial addition of H_2_O_2_, 5 ml of hydrogen peroxide was added at one-minute intervals for 10 min, and the flask was shaken for the entire duration. Once all the hydrogen peroxide was added, 4 ml of sulfuric acid (H_2_SO_4_, 98%) was added after cooling to room temperature. When the solution became transparent, 10 ml of 0.1% Tween 20 solution was added to prevent microplastics from sticking to the glass wall. After digestion, the solution was vacuum filtered using a silicon filter (10 mm × 10 mm square filters, pore diameter 17 μm, Smartmembrane). The flask was washed at least three times with filtered ultrapure water.

### Fourier-transform Infrared (FT-IR) Spectroscopy Analysis

Samples were measured by FT-IR spectroscopy on an FT-IR microscope (LUMOS II, Bruker Optics, USA), equipped with a 32 × 32 pixel Focal Plane Array detector. IR images were measured in transmission mode at a spectral resolution of 12 cm^−1^ within a spectral range from 4000 to 700 cm^−1^ and 1 scan. Before IR imaging, a photograph of the samples was taken in order to visualize surface morphology. Data analysis was conducted using the program siMPle, a freeware capable of fast detection of microplastic materials^90^; siMPle consists of an algorithm that compares the IR spectrum of the sample with each reference spectrum in the database, and then allocates the material with probability scores. The spectral library used in the analysis consists of a total of 14 types of spectra, including 11 types of plastics and four types of non-plastics (Table S3). The non-plastic category mainly includes proteins and cellulose, which are commonly detected during analysis in addition to plastics. Each spectrum contains at least six or more spectra depending on the type. The matching rate was determined by placing standard materials (Polyethylene, Polypropylene, Polystyrene & Polyethylene terephthalate: Federal Institute for Materials Research and Testing, Germany; Polyamide: Korea Institute of Analytical Science and Technology, Korea) on the silicon filter and selecting the matching rate that best matched the number of spiking particles and the number of measured particles for each plastic. We categorized the plastic material into different plastic groups using this siMPle program. Samples with a major diameter to minor diameter ratio of three or more were classified as fibers and all others were classified as fragments^91^. We used the major diameter of samples as the size and classified them into four classes: 20–49 μm, 50–99 μm, 100–299 μm, and 300–5,000 μm.

### Prevention of contamination

To prevent microplastic contamination, we excluded all plastic tools and used a metal scalpel and glass materials during the sampling and post-sampling process. Except for field collection and sampling, all sample preparation, pretreatment, and filtration steps in the lab were performed inside a laminar flow box (HSCV-1300, Sin-An Science Industry, Korea) to prevent contamination from indoor airborne microplastics, clothes, and tools. All solutions such as ultrapure water and chemical reagents were filtered using a metal filter (5 μm) before use. Filtered water and ethanol were used to clean all glassware prior to lab experiments. All samples were covered with aluminum foil when moved outside the laminar flow hood. To minimize any contamination of samples, the use of plastic materials was avoided whenever possible and nitrile gloves and cotton coats were used during all processing steps. In addition, together with the biological samples, we also prepared five control samples with empty glass vials using the same treatment protocols for comparison with experimental groups to identify any potential contamination during the sample collection and analysis process.

### Ethics declarations

No live bird was used in this study. All sampling process of carcasses was performed with permission from the Korean Ministry of Foreign Affairs in accordance with the ‘Act on Antarctic Activities and Protection of Antarctic Environment’ in the Republic of Korea.

### Supplementary Information

Supplementary Information.

## Data Availability

Any correspondence and/or requests for material should be addressed to C.Y.C.
