# Supplementary material for: Microplastics in gastrointestinal tracts of gentoo penguin (Pygoscelis papua) chicks on King George Island, Antarctica
Source: Sci Rep. 2023 Aug 10;13:13016. doi: 10.1038/s41598-023-39844-6 (PMC10415326; doi:10.1038/s41598-023-39844-6)
Supplement: Supplementary file 1 — Supplementary Information. [file 41598_2023_39844_MOESM1_ESM.pdf]

Supplementary Materials

for

Microplastics in gastrointestinal tracts of gentoo penguin (*Pygoscelis papua*) chicks on King George Island, Antarctica

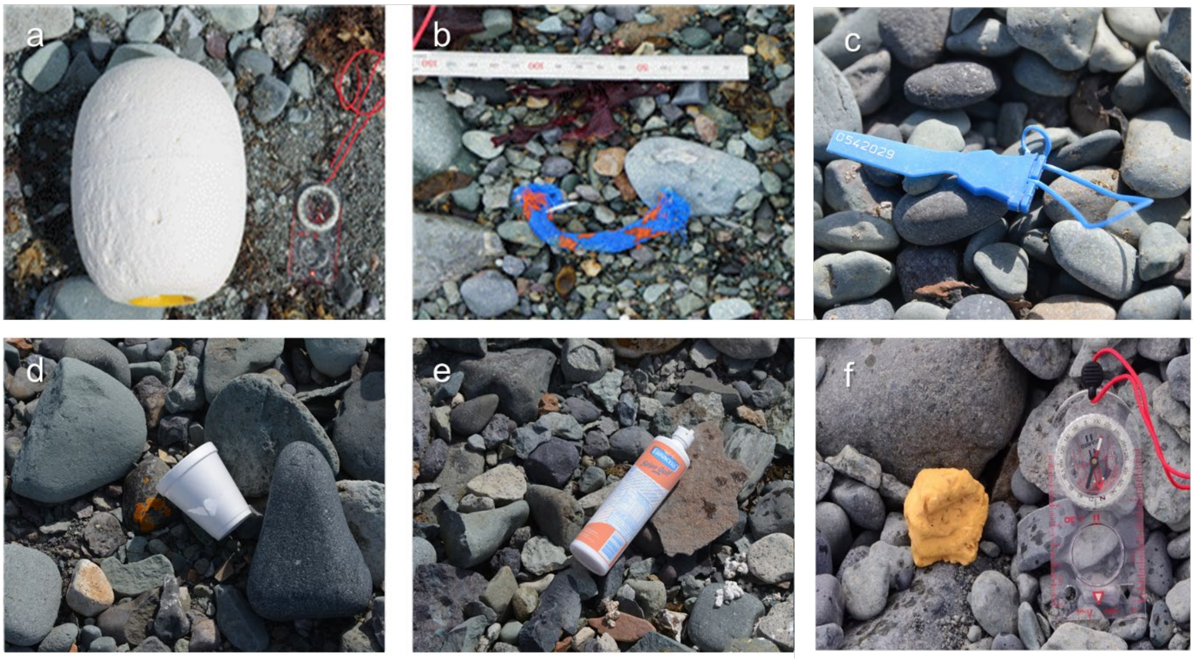

Figure S1. Plastic marine debris beached on the Barton Peninsula, King George Island, Antarctica: (a) a polystyrene buoy, (b) a piece of rope, (c) a labeling or packaging part, (d) a plastic cup, (e) a plastic bottle, and (f) a piece of polyurethane foams for insulation. All photos were taken by authors.

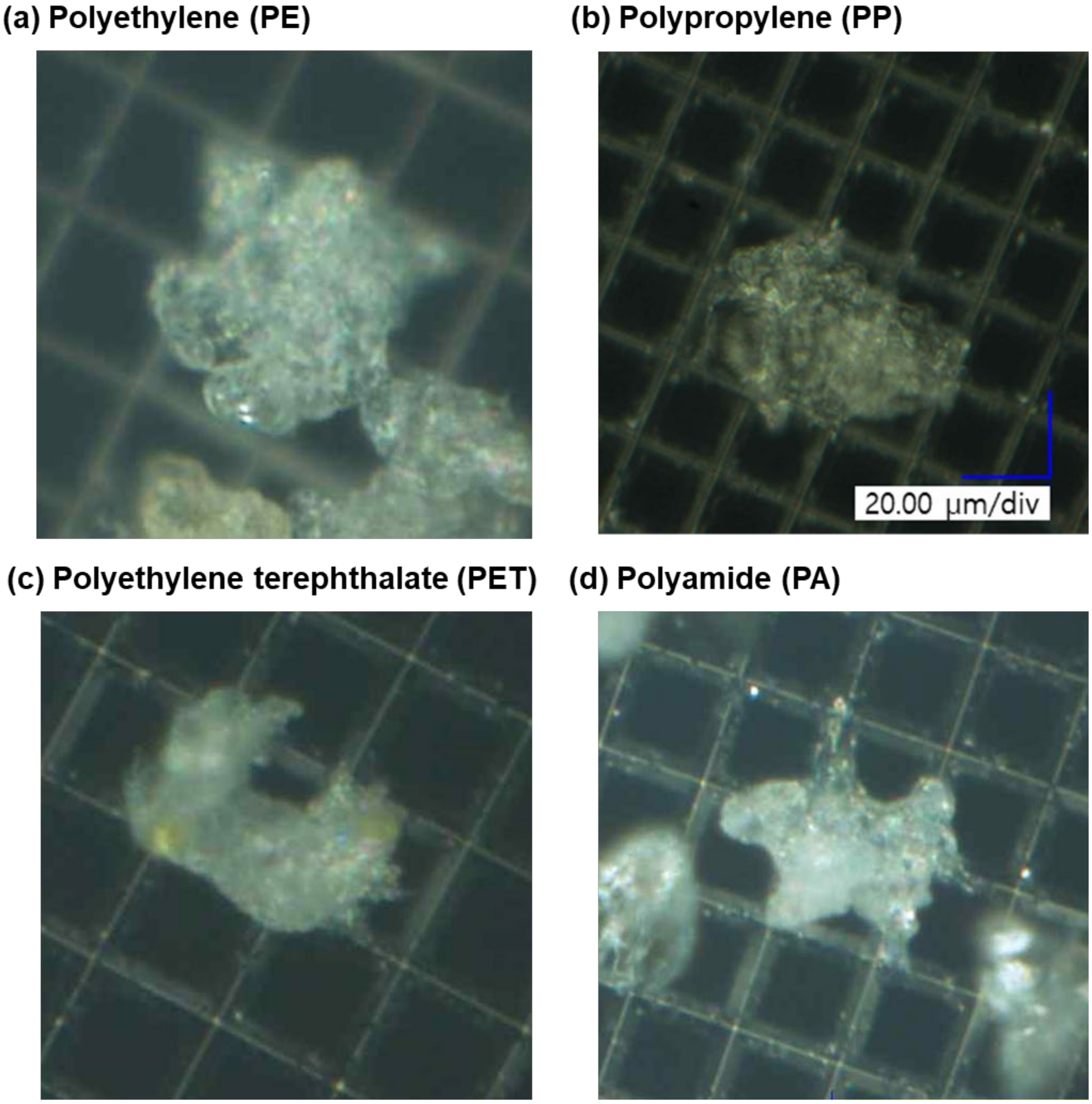

Figure S2. Microplastics found in the gastrointestinal wall and lining samples of gentoo penguin (*Pygoscelis papua*) chicks: (a) Polyethylene (PE), (b) polypropylene (PP), (b) Polyethylene terephthalate (PET), and (d) Polyamide (PA). Microplastics are shown on 20-μm silicon filters.

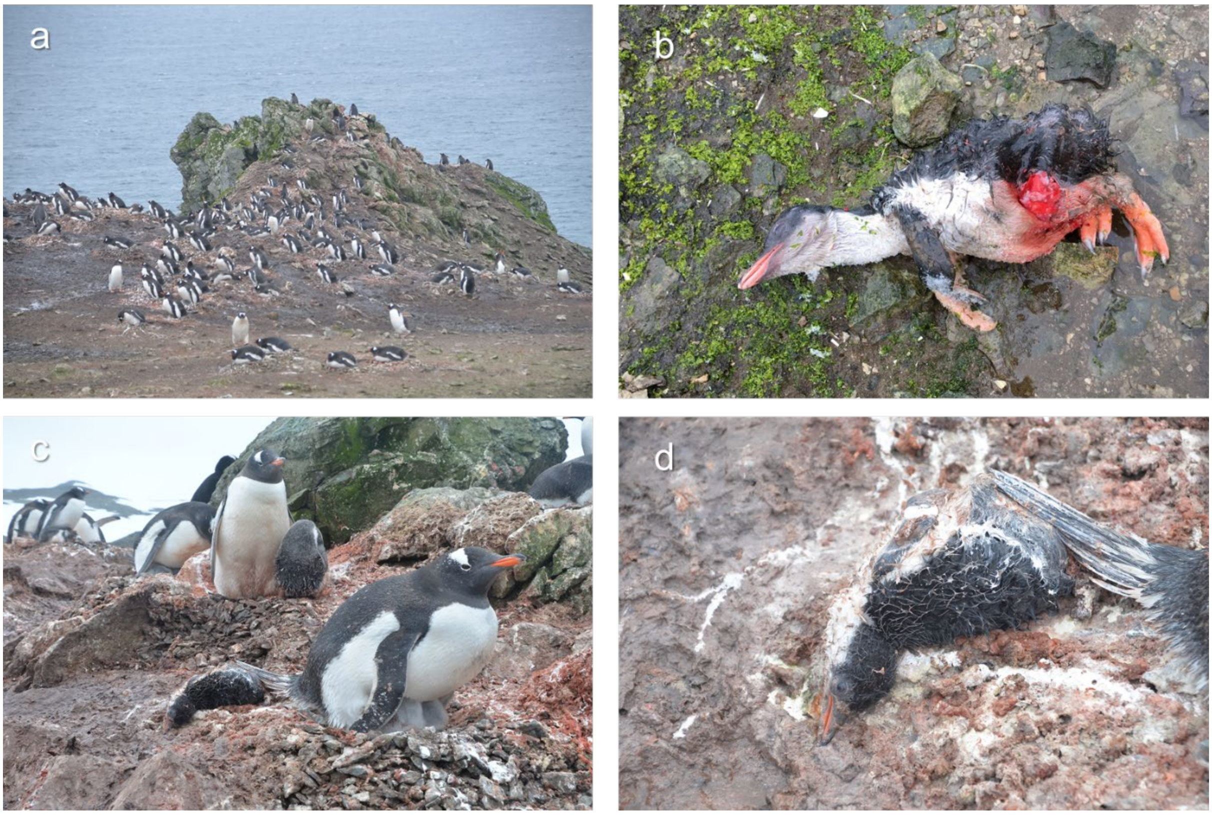

Figure S3. Gentoo penguins breeding at Narębski Point (Antarctic Specially Protected Area 171: ASPA 171) on King George Island, South Shetland Islands, Antarctica. (a) a subcolony of breeding gentoo penguins, (b) a dead chick of the gentoo penguin dragged out from its nest by a brown skua (*Stercorarius antarcticus*), (c) a dead chick on the outer lining of its nest, and (d) a closer view of the dead chick. All photos were taken by authors.

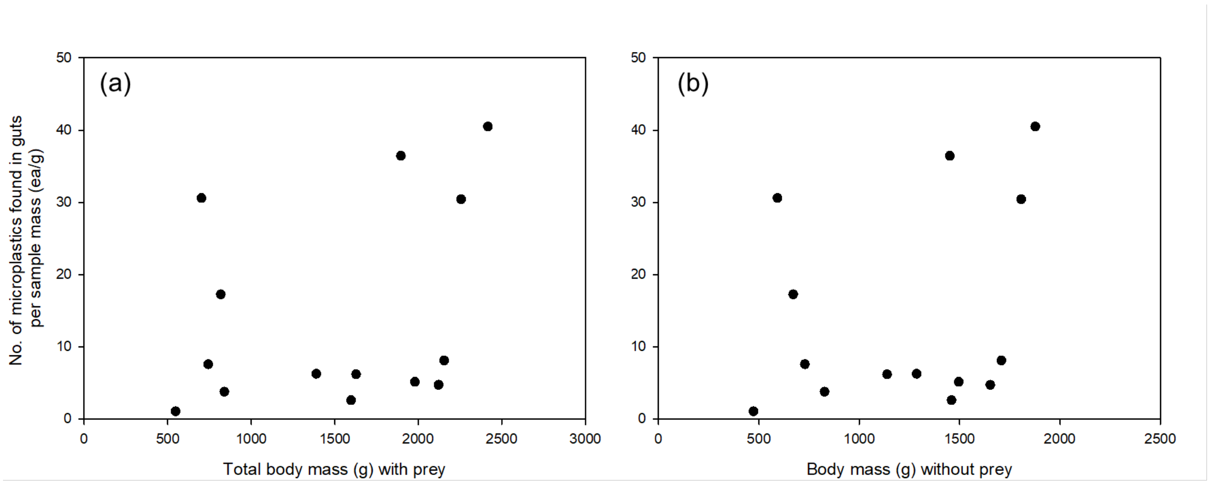

Figure S4. Scatter plot presenting the number of found microplastics per unit sample mass (1 g) and the body mass of gentoo penguins (a) with and (b) without prey in the gastrointestinal tract.

Table S1. Detail information of marine debris found during five surveys in the summer of 2013/14 and 2014/15 on the Barton Peninsula of King George Island, Antarctica

Date	Main category	Item	Types of plastic	Longitude	Latitude	Weight
2013-12-23	Metal	Metal wire	NA	-62.2383	-58.7216	0.06
2013-12-23	Others	Rubber Pad	NA	-62.2418	-58.7394	0.10
2013-12-23	Plastic	Bottle cap	Polyethylene	-62.2416	-58.7457	<0.01
2013-12-23	Plastic	PET Bottle	Polyethylene terephthalate	-62.2402	-58.7595	0.02
2013-12-23	Plastic	PET Bottle	Polyethylene terephthalate	-62.2362	-58.7174	0.02
2013-12-23	Plastic	PET Bottle	Polyethylene terephthalate	-62.2355	-58.7168	0.02
2013-12-23	Plastic	Fishing Buoy	Polyvinyl chloride	-62.2419	-58.7354	0.94
2013-12-23	Plastic	Fishing Buoy	Polyvinyl chloride	-62.2415	-58.7265	0.11
2013-12-23	Plastic	Hard Plastic	Polyethylene	-62.2416	-58.7454	<0.01
2013-12-23	Plastic	Hard Plastic	Polyethylene	-62.2427	-58.7286	<0.01
2013-12-23	Plastic	Hard Plastic	Polyethylene	-62.2383	-58.7216	0.82
2013-12-23	Plastic	Hard Plastic	Polyethylene	-62.2381	-58.7213	0.34
2013-12-23	Plastic	Plastic Straw	polypropylene	-62.2415	-58.7473	<0.01
2013-12-23	Plastic	Plastic Straw	polypropylene	-62.2427	-58.7286	0.02
2013-12-23	Plastic	Plastic bag	Polyethylene	-62.2379	-58.7679	<0.01
2013-12-23	Plastic	Insulation	Polyurethane	-62.2340	-58.7820	<0.01
2013-12-23	Plastic	Insulation	Polyurethane	-62.2342	-58.7820	<0.01
2013-12-23	Plastic	Insulation	Polyurethane	-62.2416	-58.7434	<0.01
2013-12-23	Plastic	Insulation	Polyurethane	-62.2416	-58.7430	<0.01
2013-12-23	Plastic	Insulation	Polyurethane	-62.2419	-58.7348	<0.01
2013-12-23	Plastic	Insulation	Polyurethane	-62.2415	-58.7265	<0.01
2013-12-23	Plastic	Insulation	Polyurethane	-62.2411	-58.7253	<0.01
2013-12-23	Plastic	Insulation	Polyurethane	-62.2381	-58.7211	<0.01
2013-12-23	Plastic	Rope	Polyamide	-62.2419	-58.7345	<0.01
2013-12-23	Plastic	Rope	Polyamide	-62.2341	-58.7821	0.50
2013-12-23	Plastic	Rope	Polyamide	-62.2324	-58.7121	0.34
2013-12-23	Plastic	Rope	Polyamide	-62.2392	-58.7230	0.02
2014-01-09	Metal	Can fragment	NA	-62.2419	-58.7269	<0.01
2014-01-09	Metal	Container fragments	NA	-62.2381	-58.7213	<0.01
2014-01-09	Metal	Hinge	NA	-62.2391	-58.7638	0.05
2014-01-09	Metal	Pipe	NA	-62.2417	-58.7420	0.98
2014-01-09	Metal	Metal wire	NA	-62.2361	-58.7176	0.16
2014-01-09	Metal	Ventilation cover mesh	NA	-62.2340	-58.7125	3.72
2014-01-09	Metal	Wire mesh fragment	NA	-62.2322	-58.7119	<0.01
2014-01-09	Metal	Wire mesh fragment	NA	-62.2324	-58.7121	0.52
2014-01-09	Others	Tile fragments	NA	-62.2293	-58.7087	<0.01
2014-01-09	Others	Float fragment	NA	-62.2419	-58.7476	0.18
2014-01-09	Plastic	Container fragments	Polyethylene	-62.2324	-58.7121	<0.01
2014-01-09	Plastic	Container fragments	Polyethylene	-62.2324	-58.7121	<0.01
2014-01-09	Plastic	Container fragments	Polyethylene	-62.2329	-58.7123	<0.01
2014-01-09	Plastic	Container fragments	Polyethylene	-62.2340	-58.7125	<0.01
2014-01-09	Plastic	Container fragments	Polyethylene	-62.2373	-58.7195	<0.01
2014-01-09	Plastic	Container fragments	Polyethylene	-62.2381	-58.7213	1.74
2014-01-09	Plastic	Container fragments	Polyethylene	-62.2417	-58.7420	<0.01
2014-01-09	Plastic	Container cap	Polyethylene	-62.2381	-58.7213	<0.01
2014-01-09	Plastic	Bucket fragments	Polyethylene	-62.2356	-58.7168	0.92
2014-01-09	Plastic	Bucket cap	Polyethylene	-62.2278	-58.7068	0.25
2014-01-09	Plastic	Bucket	Polyethylene	-62.2280	-58.7070	1.71
2014-01-09	Plastic	Bucket	Polyethylene	-62.2280	-58.7070	1.71

2014-01-09	Plastic	Bottle fragment	Polyethylene	-62.2419	-58.7269	<0.01
2014-01-09	Plastic	Bottle cap	Polyethylene	-62.2411	-58.7256	<0.01
2014-01-09	Plastic	Bottle	Polyethylene terephthalate	-62.2289	-58.7081	0.14
2014-01-09	Plastic	Styrofoam	Polystyrene	-62.2322	-58.7829	<0.01
2014-01-09	Plastic	Styrofoam	Polystyrene	-62.2386	-58.7648	0.03
2014-01-09	Plastic	Cushioning material	Polyethylene	-62.2381	-58.7213	<0.01
2014-01-09	Plastic	Cushioning material	Polyethylene	-62.2371	-58.7193	0.03
2014-01-09	Plastic	Sheet fragment	Polyethylene	-62.2411	-58.7256	<0.01
2014-01-09	Plastic	Plastic straw	polypropylene	-62.2311	-58.7104	<0.01
2014-01-09	Plastic	Non-fishing net	Polypropylene	-62.2280	-58.7070	0.06
2014-01-09	Plastic	Ventilation frame	Polyvinyl chloride	-62.2296	-58.7094	0.28
2014-01-09	Plastic	Ventilation cover mesh	Polyvinyl chloride	-62.2291	-58.7085	1.18
2014-01-09	Plastic	Ventilation cover mesh	Polyvinyl chloride	-62.2289	-58.7081	0.64
2014-01-09	Plastic	Ventilation cover mesh	Polyvinyl chloride	-62.2304	-58.7098	0.52
2014-01-09	Plastic	Pipe cap	Polyvinyl chloride	-62.2296	-58.7094	0.08
2014-01-09	Plastic	Pipe fragment	Polyvinyl chloride	-62.2419	-58.7269	0.15
2014-01-09	Plastic	PVC pipe	Polyvinyl chloride	-62.2351	-58.7154	0.05
2014-01-09	Plastic	Wrapping bag	Polyethylene	-62.2419	-58.7269	<0.01
2014-01-09	Plastic	Insulation	Polyurethane	-62.2279	-58.7068	<0.01
2014-01-09	Plastic	Insulation	Polyurethane	-62.2381	-58.7213	<0.01
2014-01-09	Plastic	Insulation	Polyurethane	-62.2381	-58.7213	<0.01
2014-01-09	Plastic	Insulation	Polyurethane	-62.2381	-58.7213	<0.01
2014-01-09	Plastic	Insulation	Polyurethane	-62.2411	-58.7256	<0.01
2014-01-09	Plastic	Insulation	Polyurethane	-62.2285	-58.7075	0.1
2014-01-09	Plastic	Insulation	Polyurethane	-62.2289	-58.7081	0.1
2014-01-09	Plastic	Rope	Polyamide	-62.2351	-58.7154	<0.01
2014-01-09	Plastic	Rope	Polyamide	-62.2407	-58.7529	2.74
2014-01-09	Plastic	Rope	Polyamide	-62.2322	-58.7829	0.76
2014-01-09	Plastic	Rope	Polyamide	-62.2324	-58.7121	0.16
2014-01-09	Plastic	Rope	Polyamide	-62.2381	-58.7213	0.09
2014-01-09	Plastic	Rope	Polyamide	-62.2283	-58.7072	0.07
2014-01-09	Plastic	Rope	Polyamide	-62.2329	-58.7123	0.02
2014-01-09	Plastic	Rope	Polyamide	-62.2340	-58.7125	0.02
2014-01-09	Plastic	Rope & stake	Polypropylene	-62.2373	-58.7195	0.92
2014-12-22	Others	Lemon	NA	62.2348	58.7141	<0.01
2014-12-22	Others	Lemon	NA	62.2356	58.7168	<0.01
2014-12-22	Others	Onion	NA	62.2320	58.7116	<0.01
2014-12-22	Others	Pineapple	NA	62.2350	58.7153	<0.01
2014-12-22	Plastic	Styrofoam	Polystyrene	62.2344	58.7128	<0.01
2014-12-22	Plastic	Styrofoam	Polystyrene	62.2345	58.7132	<0.01
2014-12-22	Plastic	Styrofoam	Polystyrene	62.2350	58.7152	<0.01
2014-12-22	Plastic	Styrofoam	Polystyrene	62.2417	58.7267	<0.01
2014-12-22	Plastic	Styrofoam	Polystyrene	62.2401	58.7608	<0.01
2014-12-22	Plastic	Styrofoam	Polystyrene	62.2400	58.7617	<0.01
2014-12-22	Plastic	Styrofoam	Polystyrene	62.2305	58.7855	<0.01
2014-12-22	Plastic	Styrofoam	Polystyrene	62.2289	58.7877	<0.01
2014-12-22	Plastic	Styrofoam	Polystyrene	62.2271	58.7901	<0.01
2014-12-22	Plastic	Fishing buoy	Polyvinyl chloride	62.2397	58.7239	0.54
2014-12-22	Plastic	Plastic	NA	62.2313	58.7108	0.2
2014-12-22	Plastic	Rope	Polyamide	62.2425	58.7294	<0.01
2014-12-22	Plastic	Rope	Polyamide	62.2384	58.7653	<0.01
2014-12-22	Plastic	Rope	Polyamide	62.2296	58.7869	1.2
2014-12-22	Plastic	Rope	Polyamide	62.2260	58.7919	0.58
2014-12-22	Plastic	Vynil	Polyethylene	62.2266	58.7908	<0.01
2014-12-30	Metal	Wire	NA	62.2355	58.7167	0.8

2014-12-30	Others	Lemon	NA	62.2349	58.7150	<0.01
2014-12-30	Others	Spring onion	NA	62.2355	58.7167	<0.01
2014-12-30	Others	Box,tape	NA	62.2339	58.7123	<0.01
2014-12-30	Others	Paper	NA	62.2408	58.7528	<0.01
2014-12-30	Others	Bamboo pole	NA	62.2421	58.7369	<0.01
2014-12-30	Plastic	Cap	Polyethylene	62.2418	58.7269	<0.01
2014-12-30	Plastic	Styrofoam	Polystyrene	62.2301	58.7096	<0.01
2014-12-30	Plastic	Styrofoam	Polystyrene	62.2337	58.7121	<0.01
2014-12-30	Plastic	Styrofoam	Polystyrene	62.2383	58.7214	<0.01
2014-12-30	Plastic	Styrofoam	Polystyrene	62.2386	58.7219	<0.01
2014-12-30	Plastic	Styrofoam	Polystyrene	62.2418	58.7399	<0.01
2014-12-30	Plastic	Styrofoam	Polystyrene	62.2398	58.7626	<0.01
2014-12-30	Plastic	Styrofoam	Polystyrene	62.2392	58.7636	<0.01
2014-12-30	Plastic	Styrofoam	Polystyrene	62.2329	58.7823	<0.01
2014-12-30	Plastic	Styrofoam	Polystyrene	62.2304	58.7855	<0.01
2014-12-30	Plastic	Plastic	NA	62.2349	58.7150	<0.01
2014-12-30	Plastic	Plastic	NA	62.2425	58.7294	0.54
2014-12-30	Plastic	Plastic bag	Polyethylene	62.2425	58.7295	<0.01
2014-12-30	Plastic	Rope	Polyamide	62.2319	58.7117	<0.01
2014-12-30	Plastic	Rope	Polyamide	62.2388	58.7643	<0.01
2014-12-30	Plastic	Bending rope	Polypropylene	62.2318	58.7114	<0.01
2014-12-30	Plastic	Bending rope	Polypropylene	62.2293	58.7088	<0.01
2015-01-11	Metal	Steel	NA	62.2399	58.7621	0.15
2015-01-11	Others	Cloth	NA	62.2389	58.7224	<0.01
2015-01-11	Others	Lemon	NA	62.2409	58.7251	<0.01
2015-01-11	Others	Lemon	NA	62.2407	58.7533	<0.01
2015-01-11	Others	Paper	NA	62.2401	58.7245	<0.01
2015-01-11	Others	Paper	NA	62.2403	58.7249	<0.01
2015-01-11	Others	Tissue	NA	62.3104	58.7251	<0.01
2015-01-11	Plastic	Cap	Polyethylene	62.2305	58.7097	<0.01
2015-01-11	Plastic	Pet bottle	Polyethylene terephthalate	62.2307	58.7098	<0.01
2015-01-11	Plastic	Styrofoam	Polystyrene	62.2361	58.7176	<0.01
2015-01-11	Plastic	Styrofoam	Polystyrene	62.2401	58.7245	<0.01
2015-01-11	Plastic	Styrofoam	Polystyrene	62.2405	58.7561	<0.01
2015-01-11	Plastic	Plastic	NA	62.2359	58.7173	<0.01
2015-01-11	Plastic	Tag	Polyethylene	62.2382	58.7214	<0.01
2015-01-11	Plastic	Lighter	Polyoxymethylene	62.2377	58.7199	<0.01
2015-01-11	Plastic	Pole	Polyvinyl chloride	62.2336	58.7122	0.26
2015-01-11	Plastic	Pole	Polyvinyl chloride	62.2359	58.7173	0.18
2015-01-11	Plastic	Rope	Polyamide	62.2404	58.7250	<0.01
2015-01-11	Plastic	Rope	Polyamide	62.2404	58.7250	<0.01
2015-01-11	Plastic	Rope	Polyamide	62.2408	58.7252	<0.01
2015-01-11	Plastic	Vynil	Polyethylene	62.2403	58.7249	<0.01

Table S2. Detail information of detected microplastics in the gastrointestinal wall and lining of gentoo penguin (*Pygoscelis papua*) chicks. The number of microplastics by size, form, and type in samples and control are indicated.

Source of microplastics	Size of microplastics	The number of overall microplastics (the number of microfiber)				
		Polyethylene	Polypropylene	Polyethylene terephthalate	Polyamide	Total
Sample	20-49 μm	46 (1)	22 (1)	8	4	80 (2)
	50-99 μm	94 (10)	53 (9)	18 (6)	2	167 (25)
	100-299 μm	84 (26)	36 (9)	5 (4)	-	125 (39)
	300-5,000 μm	5 (3)	-	1 (1)	-	6 (4)
Control	20-49 μm	3	2 (1)	-	-	5 (1)
	50-99 μm	1	-	-	1 (1)	2 (1)
	100-299 μm	1 (1)	4 (3)	-	-	5 (4)
	300-5,000 μm	-	-	3 (3)	-	3 (3)

Table S3. Information from the library used by siMPle (spectrum type, number, and matching rate)

Type			Basic spectrum	*Added spectrum	Total	Matching rate	
						1st range	2nd range
Plastics	Polyethylene (PE)		5 ea	6 ea	11 ea	**0.55**	**0.30**
	Polypropylene (PP)		10 ea	9 ea	19 ea	**0.50**	**0.35**
	Polystyrene (PS)		5 ea	12 ea	17 ea	**0.60**	**0.30**
	Poly(ethylene terephthalate) (PET)		5 ea	10 ea	15 ea	**0.60**	**0.30**
	Poly(vinyl chloride) (PVC)		5 ea	7 ea	12 ea	0.60	0.40
	Polyamide (PA)		5 ea	8 ea	13 ea	**0.60**	**0.30**
	Polycarbonate (PC)		5 ea	5 ea	10 ea	0.60	0.40
	Polyurethane (PU)		5 ea	3 ea	8 ea	0.60	0.40
	Poly(methyl methacrylate) (PMMA)		5 ea	10 ea	15 ea	0.60	0.40
	Polytetrafluoroethylene (PTFE)		5 ea	5 ea	10 ea	0.60	0.40
	Acrylonitrile butadiene styrene (ABS)		10 ea	-	10 ea	0.60	0.40
Non-plastics	Protein	Wool	6 ea	-	6 ea	0.60	0.40
		Skin	7 ea	-	7 ea	0.60	0.40
		Silk	6 ea	-	6 ea	0.60	0.40
	Cellulose	Cotton	7 ea	-	7 ea	0.60	0.40

* Added spectrum: Spectrum measured by transmission after placing plastic particles on silicon filters.
